# Reduced wrist flexor H-reflex excitability is linked with increased wrist proprioceptive error in adults with cerebral palsy

**DOI:** 10.3389/fneur.2022.930303

**Published:** 2022-08-09

**Authors:** S. Shekar Dukkipati, Sarah J. Walker, Michael P. Trevarrow, Morgan Busboom, Sarah E. Baker, Max J. Kurz

**Affiliations:** ^1^Boys Town National Research Hospital, Omaha, NE, United States; ^2^College of Medicine, University of Nebraska Medical Center, Omaha, NE, United States; ^3^School of Medicine, Creighton University, Omaha, NE, United States

**Keywords:** H-reflex, upper extremity, spinal cord, sensory, 1A afferent

## Abstract

Although most neurophysiological studies of persons with cerebral palsy (CP) have been focused on supraspinal networks, recent evidence points toward the spinal cord as a central contributor to their motor impairments. However, it is unclear if alterations in the spinal pathways are also linked to deficits in the sensory processing observed clinically. This investigation aimed to begin to address this knowledge gap by evaluating the flexor carpi radialis (FCR) H-reflex in adults with CP and neurotypical (NT) controls while at rest and during an isometric wrist flexion task. The maximal H-wave (Hmax) and M-wave (Mmax) at rest were calculated and utilized to compute Hmax/Mmax ratios (H:M ratios). Secondarily, the facilitation of the H-wave was measured while producing an isometric, voluntary wrist flexion contraction (i.e., active condition). Finally, a wrist position sense test was used to quantify the level of joint position sense. These results revealed that the adults with CP had a lower H:M ratio compared with the NT controls while at rest. The adults with CP were also unable to facilitate their H-reflexes with voluntary contraction and had greater position sense errors compared with the controls. Further, these results showed that the adults with CP that had greater wrist position sense errors tended to have a lower H:M ratio at rest. Overall, these findings highlight that aberration in the spinal cord pathways of adults with CP might play a role in the sensory processing deficiencies observed in adults with CP.

## Introduction

Cerebral palsy (CP) is the most prevalent pediatric neurologic impairment diagnosed in the United States (1). While the initial brain insult noted in individuals with CP is not progressive, the downstream effects on overall function can be observed over the lifespan (2). The focus of the clinical research and care of individuals with CP has primarily had an emphasis on childhood and less attention on the transition of these children into adulthood. Only four percent of all National Institutes of Health-funded research on CP between 2001 and 2013 was on adults (3). The neurophysiological changes, that are likely contributing to declining sensorimotor function as youth with CP transition into adulthood, are now receiving greater attention.

Although most of these neurophysiological studies have been focused on supraspinal networks (4–6), recent evidence points toward the spinal cord neural populations as a significant nexus between the brain and musculoskeletal components that are contributing to the extent of the gait, selective motor control, and sensory deficits seen in adults with CP (7–9). Over 50% of people living with CP report sensory deficits (10), including deficits in proprioception (11). Proprioception is thought to play a significant role in motor skill development and the production of movement (12). In adults with CP, proprioceptive deficits have been noted in several studies (13–15). Nevertheless, the sensory contribution to both motor and overall function in people with CP remains vastly underappreciated (16) as the association between motor and sensory functions remains unclear across the literature (17).

Spinal cord changes in people with CP have been suspected for at least several decades. An autopsy examination revealed clear damage to the anterior horn cells of the spinal cord, particularly at the cervical levels (18). Evidence of possible spinal motor neuron loss has also been reported based on the decreased number of motor units found in ulnar hand muscles of adults with CP (19). More recently, a structural MR imaging study of the cervical spinal cord in adults with CP uncovered anatomical reductions in the amount of spinal cord gray and white matter (20, 21). Furthermore, a reduction in the spinal cord cross-sectional area was found to be linked with the extent of the upper extremity impairment. While there is accumulating evidence that points toward changes in the number of cervical spinal motoneurons in people with CP, it is unclear how these changes may contribute to the sensorimotor integration that occurs at the spinal cord level.

Spinal inhibition of sensory pathways is depressed in patients with spasticity (22), including adults with CP (23). In particular, the post-activation depression of the Hoffman reflex (H-reflex) is diminished in adults with CP and linked with spasticity in this patient population (7). The H-reflex is a well-studied and highly replicated neurophysiological probe of the proprioceptive sensory pathways that activate the spinal motoneurons (24). It is a response that examines the overall reflex arc, including the 1A sensory afferent strength and the spinal motoneuron excitability state. Processing of the information provided by these sensory afferents allows humans to sense their bodies in space (25). Only a few studies have evaluated the amplitude of the H-reflex in individuals with CP. Collectively, these studies have shown that the soleus muscle H-reflex is increased compared to neurotypical (NT) controls (7, 26–30). However, these studies observed changes in the H-reflex by using high-frequency stimulation (>0.16 Hz), so it is unclear whether these changes were contaminated by altered post-activation depression of the responses.

In addition, while H-reflexes are typically measured at rest (31), there is a large amount of evidence suggesting that these responses are dependent on the task performed and the context (32–34). Thus, measurements obtained from individuals with CP during rest may not accurately represent the facilitation or modulation of the H-reflex during movement. In NT controls, the amplitude of the H-reflex is potentiated during voluntary contraction (34, 35). This is thought to occur primarily due to increased excitability of the motoneuron pools during a contraction (31). The few investigations that have extended these neurophysiological constructs to persons with CP have shown that the soleus H-reflex is atypically heightened during gait (27, 28). Nevertheless, there is still a void in the field's understanding of how the H-reflex is modulated from resting to an active state for the upper extremity in CP. Such insights would significantly advance the understanding of the neurophysiology of the uncharacteristic upper extremity motor actions produced by persons with CP.

Damage to the thalamocortical tracts in children with CP has been linked to somatosensory deficits, including reduced proprioception and tactile function (36). Functional neuroimaging studies have also shown that altered processing in the somatosensory cortices is connected to worse sensorimotor function in people with CP (5, 37, 38). However, these investigations have neglected to consider if regulation of 1A afferent information at the spinal cord level might play a partial role in these sensory processing deficits. The H-reflex may provide a way to fill this knowledge gap as it assesses the contribution of the entire spinal cord sensorimotor arc (i.e., 1A sensory afferents, interneurons, and motoneurons). Furthermore, prior studies with NT controls have shown that changes in the H-reflex are linked with altered proprioceptive acuity (39–41), further suggesting that alterations in the H-reflex are partially connected with sensory processing.

This investigation aimed to begin to address the large knowledge gap on the sensorimotor integration at the spinal level in adults with CP and to determine the potential link with deficits in upper extremity proprioception. This aim was addressed by evaluating the flexor carpi radialis (FCR) H-reflex in adults with CP and demographically matched NT controls while at rest and during an isometric wrist flexion task. A wrist position sense test (WPST) (42) was used to quantify the level of joint position sense for each participant. It was hypothesized that the adults with CP would display a heightened H-reflex response relative to NT controls. Secondarily, it was hypothesized that the facilitation of H-reflex responses with voluntary contraction would be reduced in adults with CP compared with the NT controls. Finally, it was hypothesized that the adults with CP would have increased position sense error relative to NT controls and that these sensory deficits would be linked with the strength of the H-reflex response.

## Materials and methods

### Participants

The Institutional Review Board reviewed and approved the protocol for this investigation (IRB #21-20-23XP). This experimental work conformed to the standards set by the Declaration of Helsinki, except for registration in a database. Participants and/or their guardians provided written informed consent to the experimental procedures and all participants assented to participate in the investigation. In total, 36 subjects participated in this investigation; 17 were adults with CP [average = 31.98 ± 11.68 yrs; 9 females; Manual Ability Classification System (MACS) levels I-IV] and 19 were NT adult controls (average = 30.81 ± 9.99 yrs; 11 females). Of the adults with CP, 10 presented with diplegia, 3 presented with right-sided hemiplegia, 2 presented with left-sided hemiplegia, and 2 presented with quadriplegia. The NT controls had no known neurological or musculoskeletal impairments that affected their hand movements at the time of the investigation. The ages of the respective groups were not significantly different (*p* > 0.05). Individuals with CP were excluded from the study if they had a dorsal root rhizotomy at the cervical levels. After the participants were enrolled in the investigation, it was observed that participants were able to exert maximal voluntary contraction (MVC) into wrist flexion with the tested limb.

### Experimental design

**Figure 1:**
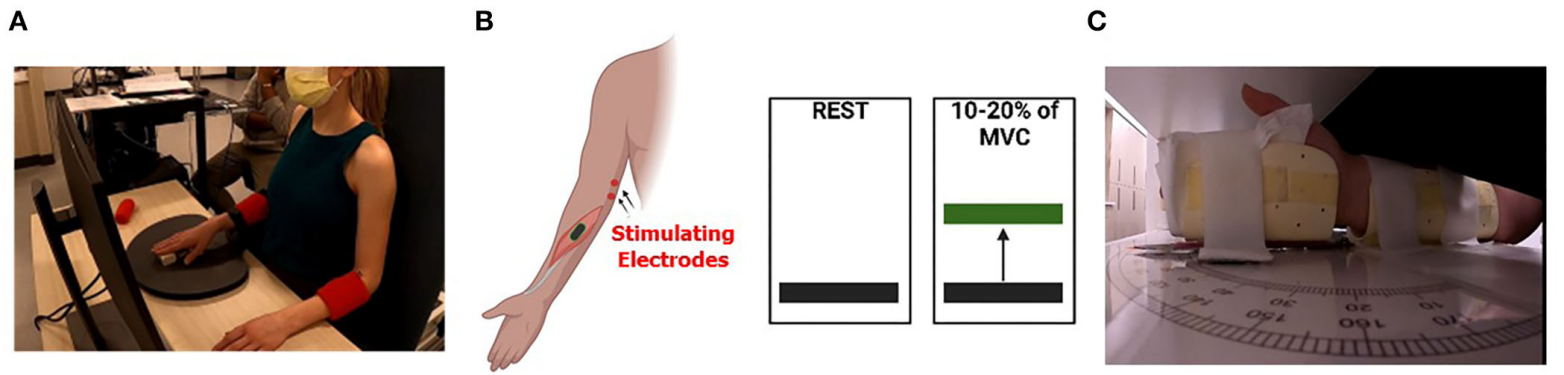
Experimental setup. **(A)** Depiction of the experimental setup where the participant is seated in a chair and viewing the wrist flexion muscular performance level on a computer screen as they attempt to match the prescribed targets. The tested arm was secured to reduce extraneous motion and the subject's wrists were in prone position. **(B)** A cartoon depicts the concept of the visual feedback displayed for subjects during the isometric wrist flexion task. Subjects were tested at rest (left) and during 10 to 20% (right) of maximal voluntary contraction (MVC). **(C)** Depiction of the positioning of the wrist within the wrist position sense test apparatus. The subject's vision of wrist position was occluded, and a black pointer located on top of the apparatus (not shown) was used by the participant to indicate the perceived wrist position.

All participants were comfortably seated in a custom, upright neck-supporting chair as shown in Figure 1A. The examined arm was positioned with the shoulder in a neutral position and the elbow flexed at 90°. The forearm of the tested arm was pronated and restrained by straps to limit compensatory movements and to isolate wrist flexion. The experimental tasks were performed with the right arm. We assumed that individuals with a diplegic presentation would have similar impairments in both arms. However, it was unclear whether this would be the case in participants with hemiplegic presentation. For the two subjects with left-sided hemiplegia, the left arm was tested. The overall study landscape consists of the participant initially undergoing an upper extremity spinal H-reflex protocol to quantify the H-reflex and maximal M-wave (Mmax) recruitment curves for the flexor carpi radialis (FCR) at rest. This was followed by having the participant perform an isometric maximal voluntary contraction (MVC) with the wrist flexors. The amplitude of the FCR electromyography during the MVC was used to set the target force levels that were used in the voluntary contraction portion of the experimental paradigm. The experiment consisted of the participants undergoing stimulation of the FCR H-reflex at their H-max while at rest (Resting Condition) and while producing an isometric wrist flexion at 10 to 20% of their MVC (Active Condition). Finally, the participants underwent a clinical assessment of their wrist position sense. Further details on the methodology employed in this investigation are detailed in the following sections.

### Electromyographic recordings

Electromyography (EMG) was recorded from the FCR muscle through surface electrodes taped to the skin over the muscle (Trigno; Delsys, Natick, MA, USA). The placement of the EMG sensors (at one-third of the distance between the medial epicondyle and radial styloid) was done with respect to anatomical landmarks and photographed to ensure similar electrode positions between subjects. To optimize the quality of the EMG signal, the skin over the FCR muscle was cleaned with 70% isopropyl alcohol prep pads before electrode placement. The collected signals were amplified, band-pass filtered (20–500 Hz), and sampled at 1,926 Hz for online and *a posteriori* analysis with a custom MATLAB program.

### Percutaneous nerve stimulation

Participants underwent an upper extremity spinal H-reflex protocol using a constant-current stimulator. Percutaneous nerve stimulation (single rectangular pulses, 1-ms pulse duration; Digitimer DS8R, Ft. Lauderdale, FL) was performed to elicit H-reflexes in the FCR muscle. To account for changes at the skin–electrode surface, maximal M-waves (Mmax) were elicited in the FCR muscle and utilized for normalization of the H-wave responses. The median nerve of the tested limb was stimulated with the unipolar surface electrodes positioned in the antecubital fossa, with the cathode placed proximally to the anode.

### Stimulus-response curves

**Figure 2:**
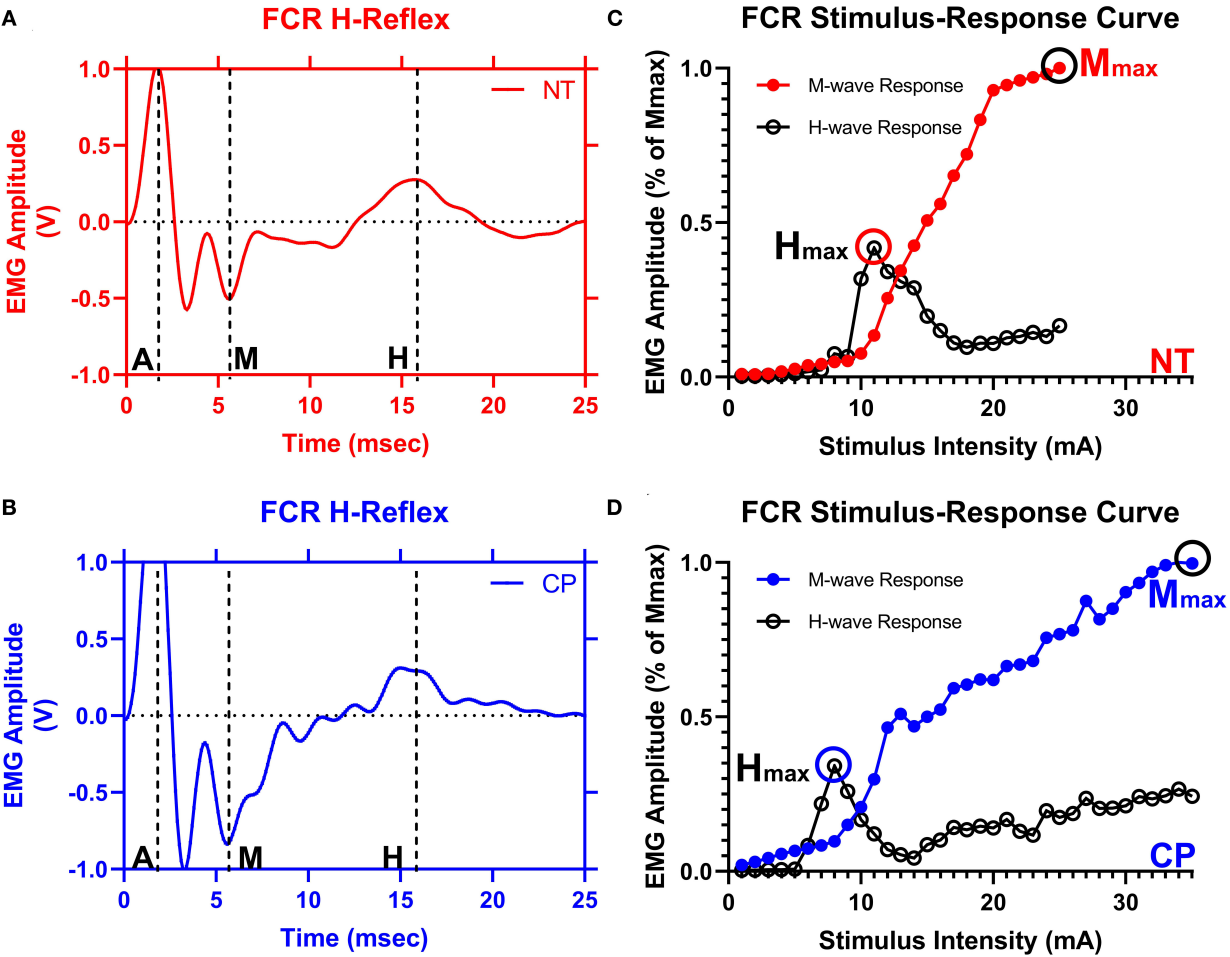
The flexor carpi radialis H-reflex is reduced in adults with cerebral palsy. Averaged H-waves and M-waves across stimulus intensities from an exemplary neurotypical (NT) adult **(A)** and an adult with cerebral palsy (CP) **(B)** are shown. The exemplary subjects were chosen based on their similarity to the means of the respective groups. EMG amplitude from the flexor carpi radialis (FCR) muscle is shown on the y-axis and time (ms) is denoted on the x-axis, with 0 ms defined as the onset of the stimulus artifact. As can be discerned in **(A,B)**, there is an M-wave response around 4 to 5 ms after the stimulus artifact and an H-wave response around 14 to 16 ms after the stimulus. M, M-wave; H, H-wave; A, stimulus artifact. **(C,D)** show M-wave (closed circle) and H-reflex (open circle) recruitment curves obtained with median nerve stimulation from the same exemplary NT control and adult with CP. Response peak-to-peak amplitudes were normalized to the maximal M-wave response (Mmax) and shown on the y-axis and stimulus intensity (in mA of current) is denoted on the x-axis. As depicted, the H-max was lower for the adult with CP when compared with the NT adult.

Stimulation with increasing levels of stimulus intensity was used to generate recruitment curves as shown in Figure 2. The Hmax and Mmax at rest were obtained from 20 to 50 stimulations, each separated by 10 s to eliminate the possibility of contamination of responses by post-activation depression. Stimulation intensity was started below the motor threshold and was sequentially increased by 1 mA increments until Mmax was elicited. Mmax was defined through visual inspection as the point beyond which M-wave amplitude no longer increased with increased current intensity. The maximal H-wave (Hmax) and the Mmax were utilized to calculate Hmax/Mmax ratios (H:M ratios).

### Active and resting task conditions

The FCR H-reflex was measured at the same relative intensity (at an intensity at or near the Hmax) during rest (Resting Condition) and a contraction of 10 to 20% of the MVC (Active Condition). For the Resting Condition, 10 H-reflexes with a stimulus interval of 10 s (ISI = 10 s) were collected while the participant sat quietly. For the Active Condition, the participants initially performed four brief MVC with the wrist flexors, separated by a minimum of 15 s of rest. The EMG data were used to quantify the magnitude of FCR activity during the MVC. EMG signals were then full-wave rectified and a zero-lag fourth-order Butterworth filter at 5 Hz was applied to generate a linear envelope (43). Peak EMG activity was subsequently determined for each participant. The rectified FCR EMG activity was shown in real-time on a computer screen and the participant was instructed to match and maintain their EMG activity at the shown target level (between 10 and 20% of the participant's MVC) for 6.5 s while they performed the isometric wrist flexion task. A between-group comparison between the background EMG levels during the contraction task showed no differences between the NT and participants with CP (*P* > 0.05). The 10 H-reflexes were collected at the respective target levels with a stimulus interval of 10 s and 3.5 s after the onset of wrist flexion (to allow ample time for all participants to reach target levels). The ratio between the mean H-reflex amplitude in the Active Condition and the mean H-reflex amplitude in the Resting Condition (Hact:Hrest) was used as the primary outcome variable to study the facilitation of H-reflexes with voluntary contraction (31).

### Wrist position sense test

The WPST quantifies the ability to indicate the joint's position following the movement performed by the examiner (Figure 1C) (42). Test stimuli included 20 predetermined wrist angles in the flexion and extension range (11 into extension and 9 into flexion). The examiner (SD) attempted to perform the passive wrist movements at a constant velocity of 10 degrees per second. The participant's vision of the wrist position was occluded by the testing apparatus. Participants indicated perceived wrist angle by aligning an arrow pointer toward the imposed wrist joint position. The perceived angle indicated by subjects was compared to the imposed angle to determine the position sense error (in degrees). Based on the prior experimental work of Carey et al. (42), the mean absolute error over the 20 positions was used as an index of proprioceptive discrimination ability (42).

### Statistical analysis

The equality of variance was initially checked for the respective outcome variables using Levene's equality of variance tests. Shapiro–Wilk testing for normal distribution revealed that these outcomes were not normally distributed within groups, as such Mann–Whitney U non-parametric testing was utilized for statistical comparisons. Finally, Spearman's correlation coefficient was performed to examine the relationship between the amount of position sense error and the spinal cord outcome measures (i.e., H:M ratios and Hact:Hrest). In all tests, statistical significance was assumed if *P* < 0.05.

## Results

Five of the adults with CP were unable to accurately match the presented targets for the Active Condition due to contractures or inadequate motor control. Their data were not included in the final comparisons to ensure that the level of muscular activity did not influence the accuracy of the Hact:Hrest calculations. Finally, one participant was unable to perform the wrist position test because their wrist was unable to be positioned appropriately to be able to complete the matching task due to a contracture limiting placement and joint mobility.

### H-reflexes at rest

**Figure 3:**
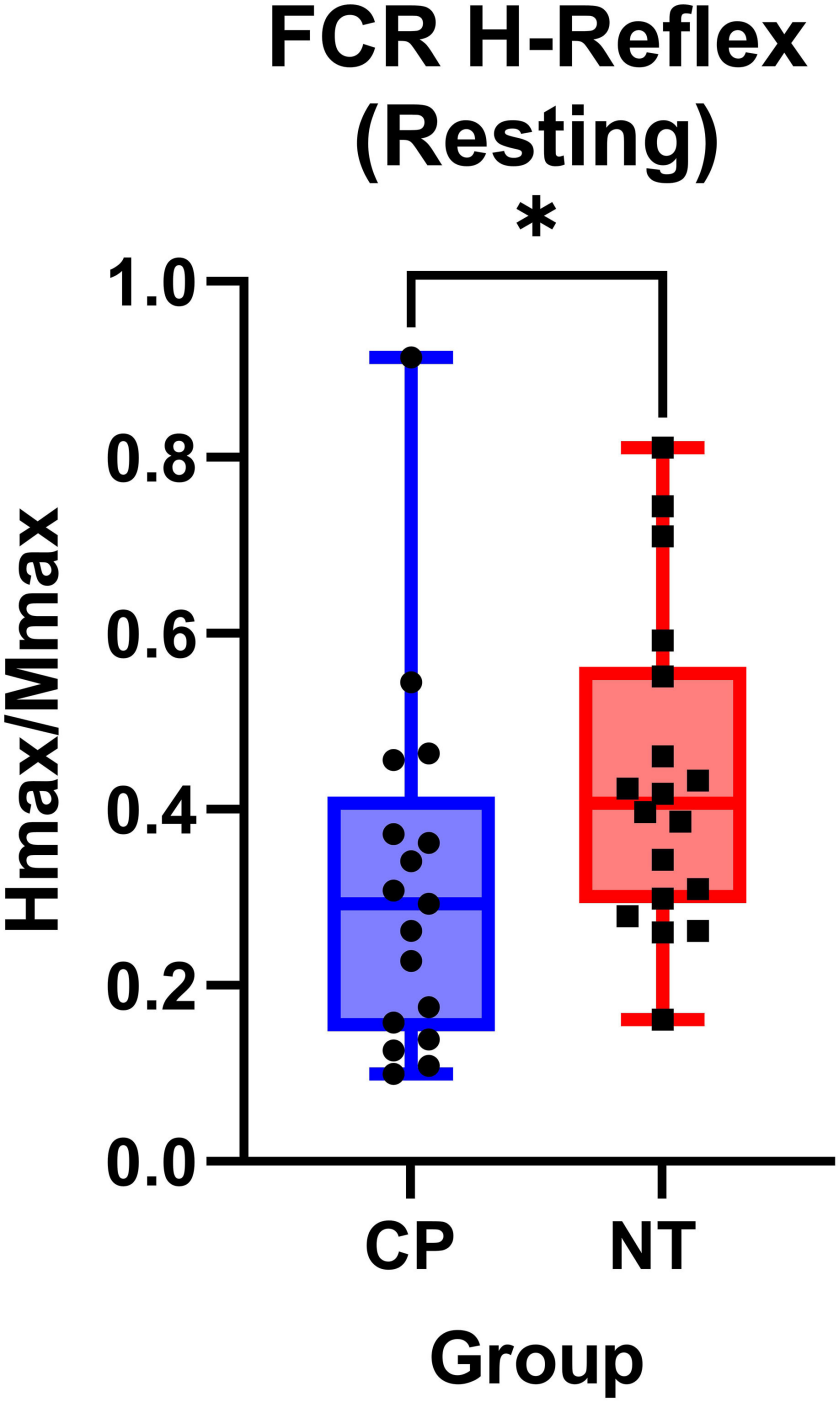
The flexor carpi radialis H:M ratio is higher in neurotypical adults compared to adults with cerebral palsy. Box-and-whisker plots of the resting flexor carpi radialis H:M ratios for neurotypical (NT) adults and adults with cerebral palsy (CP). Data are plotted with values standardized to a % of the Mmax and whiskers indicating the minimum and maximum values. As shown, the H:M ratios at rest were significantly smaller in the adults with CP, when compared with the NT controls. *Indicates *P* < 0.05.

Figure 2 illustrates examples of H waves and stimulus-response curves for a representative NT adult control (Figure 2A) and an adult with CP (Figure 2B). As the stimulus intensity was increased, the H-wave amplitude increased to a peak (Hmax) and then decreased toward a lower percentage of Mmax in both NT controls and adults with CP. Starting at higher stimulus intensities, the M-wave amplitude also increased toward a peak (Mmax) in both NT controls and adults with CP. Stimulus-response curves were generated for every participant, and the Hmax and Mmax values for each participant were utilized to calculate the H:M ratio (Hmax amplitude divided by the Mmax amplitude). Statistical analysis revealed that the adults with CP had a lower H:M ratio compared with the NT controls (CP: 0.315+/−0.205, NT: 0.436+/−0.181, *P* = 0.035). The distribution of H:M ratios (as a percentage of Mmax) for NT and adults with CP are shown in Figure 3.

### H-reflexes during voluntary contraction

**Figure 4:**
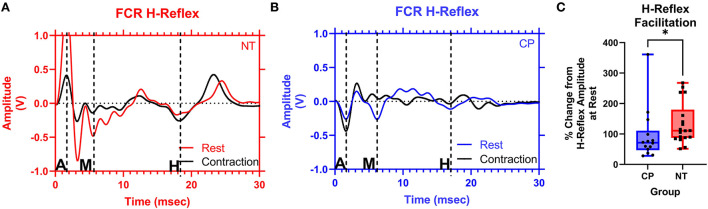
The facilitation of the flexor carpi radialis H-reflex is lower in adults with cerebral palsy than in neurotypical adults. Raw averaged flexor carpi radialis (FCR) H-waves and M-waves during rest (in red and blue, respectively) and during voluntary contraction (in black) for an exemplary NT adult control **(A)** and an adult with CP **(B)** are shown. The exemplary subjects were chosen based on their similarity to the means of the respective groups. Inspection of **(A,B)**, shows that the H-wave does not appreciably change for the adult with CP, while it is altered during the muscle contraction for the NT control. EMG amplitude from the FCR muscle is shown on the y-axis and time (ms) is denoted on the x-axis, with 0 ms defined as the onset of the stimulus artifact. M, M-wave; H, H-wave; A, stimulus artifact. **(C)** Box-and-whisker plots of the group Hact:Hrest ratios. As shown, the Hact:Hrest ratios were statistically smaller in the adults with CP, when compared with the NT controls. *Indicates *P* < 0.05.

Figure 4 illustrates examples of H waves in the FCR muscle during the Resting Condition and Active Conditions for one representative participant from each group (NT and CP). The facilitation of the H-waves during voluntary contraction was measured by obtaining the ratio between the mean H-wave amplitude during contraction (HAct) and the mean H-wave amplitude at rest (Hrest). Statistical analysis revealed that the adults with CP had less facilitation (i.e., change) of their H-reflexes with voluntary contraction compared with the NT controls (CP: 0.957+/−0.868, N = 14, NT: 1.340+/−0.689, N = 18, *P* = 0.018; Figure 4C). These results suggest that the adults with CP were unable to modulate the H-reflex while producing a motor action.

### Wrist position sense

**Figure 5:**
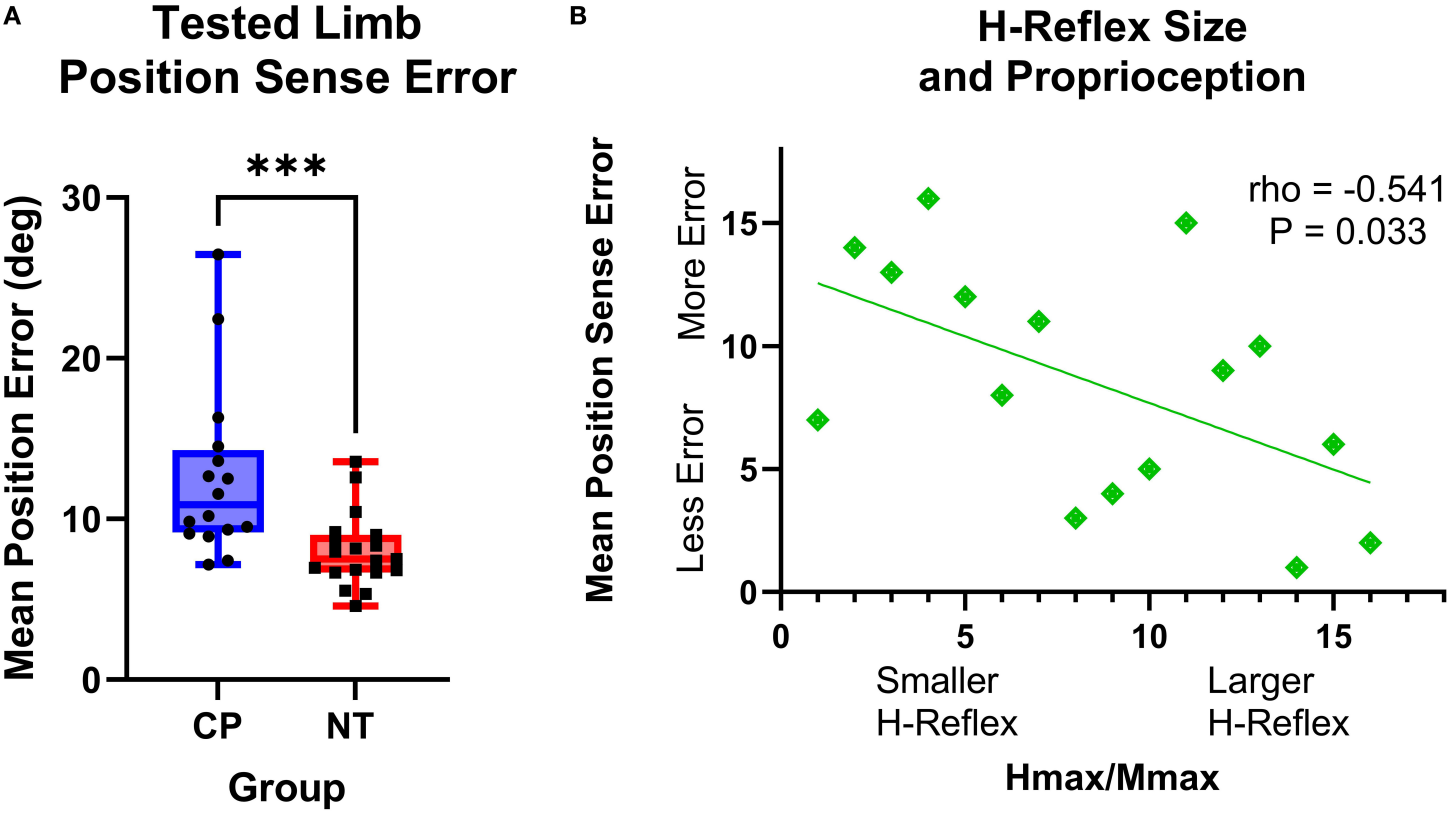
The H-reflex size is related to position sense error in adults with cerebral palsy. **(A)** Box-and-whisker plots of the group mean position errors for the adults with cerebral palsy (CP) and neurotypical (NT) adult controls. As shown, the average position sense errors were significantly greater for the adults with CP. ***Indicates *P* < 0.001. **(B)** Rank-order correlation between the mean position sense error and H-reflex H:M ratios at rest for the adults with CP. As shown, there was a significant negative correlation between the strength of the H-reflex size (H:M ratio) and the participant's mean position sense error (rho = −0.541; *P* = 0.033). This implies that adults with CP that had a lower H-reflex at rest also tended to have greater errors in their perception of wrist joint position.

The adults with CP had greater wrist position sense errors compared with the NT controls (CP: 12.59+/−5.32 degrees of error, NT: 8.01+/−2.28 degrees of error, *P* = 0.0003; Figure 5A). Furthermore, there was a significant rank-order relationship between the mean position sense error and H:M ratios at rest for the adults with CP (rho = −0.541, *P* = 0.033, rho^2^ = 29%). This correlation implies that the adults with CP who had smaller wrist flexor H-reflexes tended to have a worse perception of the wrist joint position (Figure 5B). No other correlations between position sense and H-reflex measures were significant (*P* > 0.05).

## Discussion

This study used upper extremity H-reflex testing to examine the putative changes in proprioceptive processing of 1A afferents in adults with CP at the spinal cord level during rest and as they volitionally performed an isometric wrist flexion task. Contrary to the original hypothesis, the adults with CP displayed a diminished H-reflex response relative to NT controls at rest. The hypothesis that the facilitation of H-reflex responses with voluntary contraction would be reduced in adults with CP compared with the NT controls was confirmed, as the adults with CP did not modulate their H-reflex while performing a contraction. Finally, the results also supported the hypotheses that the adults with CP would have increased position sense error relative to NT controls and that these sensory deficits would be linked with the strength of the H-reflex response. Overall, these results imply that there are deficits in sensorimotor integration at the spinal cord level in adults with CP. Further implications of these experimental results are discussed in the following sections.

### H-reflexes and motoneuron pools in CP

One of the key findings in this study is that the adults with CP had lower FCR H:M ratios when measured at rest. This is a novel finding as upper extremity H:M ratios have not been previously evaluated in this population. Rather, the scientific literature has predominantly focused on the H:M ratios of the lower extremity soleus muscle in individuals with CP and have contrarily shown that the soleus H:M ratios were increased in adults with CP (7). The differences noted here are likely related to the muscle fiber types that comprise the FCR and soleus. The FCR is a muscle with a mixed composition of higher threshold (fast-type) and lower threshold (slow-type) motor units (44, 45), which is in contrast to the large slow-type composition of the soleus in humans (46). Studies on upper limb muscle fiber types in children and young adults with CP generally describe a slow- to fast-type shift in the muscle fiber architecture (47, 48). Intriguingly, there is immunohistochemical evidence that there is a higher incidence of fast-type muscle fiber composition in the wrist flexors with more impairment in people with CP (49). This altered composition may result from the adaptation of motor unit properties resulting from a decreased amount of voluntary recruitment of these units over the lifespan. As fast-type motor units are thought to be less easily excited by the 1A afferents during nerve stimulation and may receive less muscle spindle feedback than slow-type motor units (50, 51), the attenuated FCR H-reflex responses that were observed here in adults with CP may be a marker of upper extremity motor unit remodeling. Future studies comparing muscle properties and motor unit composition with H-reflex measures in persons with CP may shed light on this possibility.

There was less facilitation of the H-reflex with voluntary contraction of the wrist flexors in adults with CP. As the size of the H-reflex is thought to increase during contraction due to increased excitability of the motoneuron pools (31), it may be postulated that the motoneuron pools that have a higher firing threshold (fast-type motoneurons) may not be as easily activated through 1A sensory afferents in adults with CP or have intrinsic properties that make them less excitable. This conjecture is plausible as prior studies have shown that individuals with CP have greater difficulty with recruiting higher threshold motor units with contraction (52).

Alternatively, the results might convey that there are fewer motoneurons available for recruitment with contraction in adults with CP. Recent structural neuroimaging studies have shown that the cervical gray matter cross-sectional area is reduced in adults with CP and this reduction is connected to both the aberrant sensorimotor cortical activity in this population and the overall level of hand motor impairment (20, 21). Thus, it is very plausible that the reduced FCR H-reflex excitability might reflect the changes in the size of the motor pools and/or interneurons that compose the gray matter at the cervical levels. Reductions in the number of spinal motoneurons have been noted in both animal models of CP (53, 54) and adult patients with CP (19). The findings of reduced H-reflex excitability do not rule out this possibility, but further studies linking motor unit number estimation and H-reflex excitability in people with CP are needed for confirmation.

### H-reflexes and proprioception in CP

The results from this study further confirmed the notion that adults with CP likely have proprioceptive errors. Past studies of children and adults with CP have identified decreased limb position sense in both the dominant and non-dominant limbs (13, 15, 55, 56). Intact proprioception is an important part of optimal motor control, allowing for the updating of internal models throughout the lifespan (12). Proprioceptive information comes to the central nervous system primarily through 1A afferents from the muscle spindles (25, 57, 58). This information is processed at the cortical level (in the primary and secondary sensorimotor cortices), the subcortical level (in the basal ganglia and cerebellum), and the spinal level (by the interneurons and motoneurons). Hence, the proprioceptive errors noted here likely contribute to the altered motor actions seen in adults with CP.

Interestingly, these results revealed that smaller H-reflexes seen at the spinal cord level are related to a higher amount of proprioceptive error in adults with CP. The H-reflex has been touted for many decades as a neurophysiological probe of the proprioceptive pathways that activate spinal motoneurons through sensory 1A afferents (24, 59, 60). Based on this premise, the smaller H-reflexes observed in adults with CP may suggest a decreased sensory afferent innervation of the spinal motoneurons and/or interneurons or a decreased strength in transmission at the 1A-motoneuron synapse. Overall, the importance of integrating proprioceptive feedback at the spinal level has been suggested but not fully appreciated (9, 61). Sensory afferent feedback activity also contributes directly to muscle activation through spinal networks, allowing spinal cord-regulated control in the task-dependent facilitation of muscle activation (62). In this study, only one component of proprioception in joint position sense was explored, but future studies exploring the connections between H-reflexes (especially during voluntary contraction) and kinesthesia in people with CP may be highly informative.

The relationship between the H-reflex size and proprioceptive acuity may also suggest that adults with CP have an altered developmental trajectory of the somatosensory systems that weakens sensory afferent activity. Perinatal lesions of the corticospinal tract (CST) are thought to contribute to the abnormal development of spinal sensory reflex pathways (63). The muscle sensory afferents which mediate proprioception coordinate the development of their spinal connections with those of the CST (64, 65). Selective CST injury has been shown to induce maladaptive sensory afferent fiber plasticity in the spinal cord in rodent models (66) and the CST projections are thought to be dynamically regulated through the lifespan by the activity-dependent actions of these sensory afferents (67). It is presumed that changes in the primary sensory afferent pathways may be related to dynamic modifications of the sensorimotor pathways that include both changes in corticospinal tract connectivity and the aberrant somatosensory and motor cortical processing often seen in persons with CP across the lifespan. These are exciting open questions that could address the contributions of different neural populations to the somatosensory deficits that not only decrease quality of life and overall function but may also contribute to decreased motor control in many persons with CP.

### Sensory function in adults with CP

The results from this study showing sensory deficits in the upper extremity across the cohort of adults with CP and a spastic diplegic presentation were somewhat surprising given that those with diplegia are assumed to exclusively have lower extremity impairments. This may be due to the strength of the WPST to distinguish deficits in position sense in clinical populations (42, 68, 69), but also may reflect an inadequate clinical picture of those with a diplegic presentation. The current findings indicate that hand proprioceptive acuity is diminished in adults with CP, building on past studies that have revealed similar deficits across the lifespan (13–15, 55, 56). However, the number of studies related to proprioception in this population remains very low (11). Further investigation of the source and timing of these sensory deficits in people with CP may reveal effective targets for therapeutic intervention, as suggested in prior studies (15, 69, 70). The relationship of these sensory deficits to the extent of the upper extremity motor control challenges in individuals with CP across the lifespan remains an important area for further study.

### Limitations

The interpretation of the connection between the processing of proprioceptive information and the H-reflex outcomes seen here is somewhat complicated by two premises. First, the H-reflex carries information about the excitability of the 1A afferents, motoneuron excitability, supraspinal modulation, and neuromodulation via persistent inward currents. Second, the 1A afferents have mono-synaptic connections with the alpha motoneurons and concomitant connections with the spinal interneurons and the higher order sensory processing centers (thalamus and somatosensory cortex). However, it has been identified that the primary source for both the H-reflex activation and the sensory information that is processed during kinesthetic tasks by the somatosensory cortices and thalamus are the 1A pathways originating from the muscle spindles (57). The hypothesis that changes in the H-reflex may be partially linked with changes in proprioceptive acuity is further supported by prior studies that have shown that vibrations applied to the muscle 1A afferents attenuate the FCR H-reflex (39, 41) and degrade the proprioceptive acuity of neurotypical controls (39, 41). In addition, hyperthermia has also been shown to reduce the H-reflex and results in complementary changes in proprioceptive error (40). Thus, there seems to be sufficient evidence that there are connections between H-reflex size and proprioceptive error.

It is also conceivable that the reduced H-reflex size noted may be due to increased presynaptic inhibition, which has been noted to be altered with modified proprioceptive feedback (71). While presynaptic inhibition of the H-reflex was not specifically assessed, the observed changes in this study may be related to changes in presynaptic inhibition. The processing of the 1A afferent information through this pathway could be responsible for the observed changes, although it is highly unlikely given the past report of decreased, not increased, pre-synaptic inhibition in people with CP. Intriguingly, Achache et al. (7) found that presynaptic inhibition was reduced in adults with CP (7), which would drive the H-reflex size to be larger, which is the opposite of what was observed in this study.

Finally, it is possible that the recording electrodes and stimulation electrodes might have slightly shifted position during the active contraction, which could lead to distortion of the muscle/recording electrode or nerve/stimulating electrode relationship during the task (51). This could have potentially been overcome by normalizing the H-reflex values to an Mmax obtained during the active task, although Mmax differences during voluntary contraction have been noted to be minimal when grouping subjects together (51). However, the Mmax during the active motor control task was not calculated because it would affect the precision of the motor task being performed and did not obtain after the task to limit the amount of supramaximal stimulation utilized in this study. Thus, it is possible that the magnitude of the H-reflex changes noted during the active task in people with CP may be impacted by altered Mmax values during voluntary contraction. In addition, not all subjects with CP were able to complete the voluntary contraction task, affecting the sample sizes of the H-reflex responses collected during the active condition. Further studies with larger sample sizes looking at the impact of voluntary contraction on both H-reflex and M-max sizes in people with CP may offer more clarity.

## Conclusion

Adults with CP appear to have reduced excitability in the wrist flexor H-reflex. These changes were seen during both rest and voluntary contraction, suggesting an impact from a diversity of motor unit types. This reduction was linked with the extent of the wrist proprioceptive impairment. Under the premise that the H-reflex partially reflects the sensory information that is processed during kinesthetic tasks (57), these findings imply that the altered somatosensory processing seen in adults with CP might partially arise due to disruptions at the spinal cord level and/or developmental changes at the motor unit level triggered by changes in descending inputs from the sensorimotor cortices. Further investigations of the spinal cord interneuron dynamics are warranted to further illuminate how alterations at the spinal cord level impact the sensory processing and motor actions of adults with CP. In addition, it is increasingly apparent that adults with CP have ongoing sensory impairment throughout the lifespan, worthy of attention beyond childhood and motor-focused approaches. Spinal cord neuromodulation targeting the spinal reflex arc may offer an appropriate avenue for investigations toward this cause.

## Data availability statement

The raw data supporting the conclusions of this article will be made available by the authors, without undue reservation.

## Ethics statement

The studies involving human participants were reviewed and approved by Boys Town IRB. The patients/participants provided their written informed consent to participate in this study.

## Author contributions

SD and MK contributed to conception and design of the study. SD, SW, MT, and MB collected the data. SD, SB, and MK contributed to the data processing protocols. SD performed the statistical analysis and wrote the first draft of the manuscript. All authors contributed to manuscript revision, read, and approved the submitted version.

## Funding

This work was supported by a grants from the National Institutes of Health (R01HD086245, R01HD101833, R21HD096390, and P20GM144641).

## Conflict of interest

The authors declare that the research was conducted in the absence of any commercial or financial relationships that could be construed as a potential conflict of interest.

## Publisher's note

All claims expressed in this article are solely those of the authors and do not necessarily represent those of their affiliated organizations, or those of the publisher, the editors and the reviewers. Any product that may be evaluated in this article, or claim that may be made by its manufacturer, is not guaranteed or endorsed by the publisher.

## Abbreviations

H-reflex: Hoffman reflex
CP: cerebral palsy
NT: neurotypical
MVC: maximal voluntary contraction
H:M ratio: maximal H wave to maximal M wave ratio
CST: corticospinal tracts.
